# Draft Genome Sequence of *Pediococcus lolii* NGRI 0510Q^T^ Isolated from Ryegrass Silage

**DOI:** 10.1128/genomeA.00156-12

**Published:** 2013-02-07

**Authors:** Katsumi Doi, Kazuki Mori, Kosuke Tashiro, Yasuhiro Fujino, Yuko Nagayoshi, Yoshiharu Hayashi, Satoru Kuhara, Toshihisa Ohshima

**Affiliations:** a Microbial Genetic Division, Institute of Genetic Resources, Faculty of Agriculture; b Department of Bioscience and Biotechnology, Faculty of Agriculture; c Division for Arts and Science, Faculty of Arts and Science, Kyushu University, Fukuoka, Japan; d Scientific Affairs, Life Technologies Japan Ltd., Tokyo, Japan

## Abstract

*Pediococcus lolii* NGRI 0510Q^T^ was isolated from ryegrass silage produced on Ishigaki Island, Okinawa Prefecture, Japan. Here we present a draft genome sequence for this strain, consisting of 103 contigs for a total of 2,047,078 bp, 2,154 predicted coding sequences, and a G+C content of 42.1%.

## GENOME ANNOUNCEMENT

The genus *Pediococcus* includes Gram-positive, catalase-negative, and oxidase-negative lactic acid bacteria (1). Most of the known pediococcal strains were isolated from various plants and fruits, fermenting vegetables, beer, and silage. Genome sequences have been published for *Pediococcus acidilactici* (2), *P. claussenii* (3), and *P. pentosaceus* (4). *P. lolii* NGRI 0510Q^T^ was isolated from ryegrass silage produced on Ishigaki Island, Okinawa Prefecture, Japan (5). Although *P. lolii* appears closely related to *P. acidilactici* and *P. pentosaceus* at the 16S rRNA sequence level, it exhibits significant phenotypic differences, such as the ability to utilize rhamnose and the inability to produce acid from trehalose. Detailed investigation of the phylogenic and taxonomic characteristics of this strain should improve our understanding of the comparative genomics of *Pediococcus* strains having diverse ecological origins and facilitate their practical application.

The *P. lolii* sample was prepared for sequencing by growing the organism anaerobically overnight at 37°C in MRS broth. Genomic DNA was then extracted and purified as described by Marmur (6) with some modifications. Bar-coded library DNAs were prepared by using an Ion Plus fragment library kit and an Ion Xpress Bar code adaptors 1-16 kit according to the manufacturer’s protocol for 200 base-read sequencing. One microgram of the product was used for preparation of each DNA library, and the libraries were amplified through 8 cycles of PCR. Sequencing was performed by using an Ion PGM 200 sequencing kit and an Ion 316 chip with a flow number of 520 for 200 base reads.

The genomic DNA, which included a total of 2,047,078 bp, was sequenced using a whole-genome shotgun strategy that generated 2,418,179 reads and attained approximately 232-fold coverage. Assembly of all the reads by using Newbler Assembler Ver. 2.7 software resulted in 103 contigs (>100 bp) with an N50 contig size of 102,322 bp. Genome annotation of the obtained scaffolds was performed by using Glimmer 3.02 software and BLAST searches against a nonredundant protein sequence database. The genome of strain NGRI 0510Q^T^ has a G+C content of 41.2%, and annotation using the GTPS (7), RDP, and Silva databases with tRNAscan-SE v.1.21 (8) and with further manual inspection revealed 2,154 predicted coding regions, 54 tRNA genes, and 2 rRNA genes.

Open reading frames (ORFs) containing rhamnose metabolite-related genes encoding l-rhamnose isomerase, rhamnulokinase, and rhamnulose-1-phosphate aldolase were annotated in the BANK01000012. Although all gene homologues have been annotated in *P. acidilactici* 7_4 and a homologue of only the rhamnulokinase gene was annotated in *P. acidilactici* DSM 20284^T^, they were not detected in the *P. acidilactici* M18/5M, *P. claussenii* ATCC BAA-344^T^, and *P. pentosaceus* ATCC 25745 genomes (NZ_AGKB01000000, GCF_000237995.1 and GCF_000014505.1). Hence, those genes likely account for the difference in rhamnose utilization between *P. lolii* and other *Pediococcus* strains. Genome analysis also revealed the presence of a putative prophage of about 38 kb containing various prophage Lp1 proteins (9) and several racemases, including alanine racemase, aspartate racemase, and glutamate racemase (10).

### Nucleotide sequence accession numbers.

The *P. lolii* NGRI 0510Q^T^ genome sequence and annotation data have been deposited in the DDBJ/EMBL/GenBank under accession numbers BANK01000001 to BANK01000103.
